# Mebendazole Treatment Disrupts the Transcriptional Activity of Hypoxia-Inducible Factors 1 and 2 in Breast Cancer Cells

**DOI:** 10.3390/cancers15041330

**Published:** 2023-02-20

**Authors:** Natalie S. Joe, Yuanfeng Wang, Harsh H. Oza, Inês Godet, Nubaira Milki, Gregory J. Riggins, Daniele M. Gilkes

**Affiliations:** 1 Department of Oncology, The Sidney Kimmel Comprehensive Cancer Center, The Johns Hopkins University School of Medicine, Baltimore, MD 21231, USA; 2 Cellular and Molecular Medicine Program, The Johns Hopkins University School of Medicine, Baltimore, MD 21231, USA; 3 Biochemistry and Molecular Biology Program, The Johns Hopkins University School of Public Health, Baltimore, MD 21205, USA; 4 Department of Chemical and Biomolecular Engineering, The Johns Hopkins University, Baltimore, MD 21218, USA; 5 Johns Hopkins Institute for NanoBioTechnology, The Johns Hopkins University, Baltimore, MD 21218, USA; 6 Department of Neurosurgery, The Johns Hopkins University School of Medicine, Baltimore, MD 21231, USA

**Keywords:** mebendazole, hypoxia, HIF1, HIF2, digoxin, breast cancer

## Abstract

**Simple Summary:**

Mebendazole (MBZ), an orally available, FDA-approved anthelmintic, has demonstrated efficacy in reducing solid tumor growth and preventing or treating metastasis in multiple preclinical models of cancer. MBZ was also well tolerated in a recently completed phase I clinical trial. Given the success of MBZ, we aimed to identify additional mechanisms of action for MBZ beyond those that have been previously reported, which include tubulin disruption, inhibiting angiogenesis, promoting apoptosis, and maintaining cell stemness. We demonstrate that MBZ can inhibit the transcriptional activity of HIFs in breast cancer cell lines and in preclinical models of breast cancer by preventing the induction of HIF-1α, HIF-2α, and HIF-1β at the protein level under hypoxic conditions. We show that MBZ treatment has dual use as a chemotherapeutic agent as well as blocking the hypoxia-induced phenotype that promotes chemoresistance.

**Abstract:**

Breast cancer is the most diagnosed cancer in women in the world. Mebendazole (MBZ) has been demonstrated to have preclinical efficacy across multiple cancers, including glioblastoma multiforme, medulloblastoma, colon, breast, pancreatic, and thyroid cancers. MBZ was also well tolerated in a recent phase I clinical trial of adults diagnosed with glioma. The mechanisms of action reported so far for MBZ include tubulin disruption, inhibiting angiogenesis, promoting apoptosis, and maintaining stemness. To elucidate additional mechanisms of action for mebendazole (MBZ), we performed RNA sequencing of three different breast cancer cell lines treated with either MBZ or vehicle control. We compared the top genes downregulated upon MBZ treatment with expression profiles of cells treated with over 15,000 perturbagens using the clue.io online analysis tool. In addition to tubulin inhibitors, the gene expression profile that correlated most with MBZ treatment matched the profile of cells treated with known hypoxia-inducible factor (HIF-1α and -2α) inhibitors. The HIF pathway is the main driver of the cellular response to hypoxia, which occurs in solid tumors. Preclinical data support using HIF inhibitors in combination with standard of care to treat solid tumors. Therefore, we tested the hypothesis that MBZ could inhibit the hypoxia response. Using RNA sequencing and HIF-reporter assays, we demonstrate that MBZ inhibits the transcriptional activity of HIFs in breast cancer cell lines and in mouse models of breast cancer by preventing the induction of HIF-1α, HIF-2α, and HIF-1β protein under hypoxia. Taken together, our results suggest that MBZ treatment has additional therapeutic efficacy in the setting of hypoxia and warrants further consideration as a cancer therapy.

## 1. Introduction

Mebendazole (MBZ) was approved by the FDA in 1971 as an anthelmintic drug used to treat parasitic infections [1]. More recently, MBZ has been shown to have preclinical efficacy [2] in treating pancreatic [3], lung [4,5], thyroid [6], breast [7], colorectal [8], and brain tumors [9,10,11]. The first known mechanism to support the efficacy of MBZ as a treatment for cancer was its ability to disrupt tubulin, a common target of chemotherapeutics [12]. Other reported tumor-suppressive mechanisms for MBZ include inhibiting angiogenesis, inducing apoptosis through BCL-2 and caspase-3-dependent mechanisms, and reducing AKT and STAT3 activity [11,13,14]. In addition, we recently showed that MBZ could also reduce the stem-like phenotype of triple-negative breast cancer cells via ITBβ4, leading to tumor shrinkage and reduced metastasis [15].

To elucidate additional signaling pathways affected by MBZ treatment, we queried the clue.io database [16], which contains differential gene expression profiles resulting from cellular perturbations, including both genetic and small molecule perturbagens. The database revealed that the expression patterns following treatment with MBZ closely matched the profile of cells treated with either angiogenesis, cell cycle, or tubulin inhibitors, which aligns with previously reported MBZ mechanisms. Surprisingly, the gene expression profile following MBZ treatment also matched the profile of cells treated with known HIF inhibitors [17], including Digoxin and Vorinostat.

A common feature of solid tumors is hypoxia, or an imbalance in O_2_ consumption and demand, which causes an overall decrease in O_2_ levels in the microenvironment [18,19]. When a cell experiences hypoxia, two transcription factors, hypoxia-inducible factors HIF-1α and/or HIF-2α, are stabilized and dimerize with HIF-1β, regulating the transcription of more than 1000 hypoxia-inducible genes [20]. In the context of cancer, hypoxia has been linked to a higher risk of metastases and mortality [21]. In addition, key genes that promote chemoresistance, altered metabolism, invasion, metastases, cell proliferation, and angiogenesis are regulated by HIFs under hypoxic conditions [20].

The results led us to hypothesize that MBZ may suppress HIF activity and, thus, be effective at promoting the eradication of hypoxic cells that are less sensitive to chemotherapy [22,23]. Using RNA sequencing and luciferase reporter assays, we demonstrate that MBZ can inhibit the transcriptional activity of HIF target genes. We further show that MBZ decreases HIF-1α, HIF-2α, and HIF-1β at the protein level under hypoxia but does not alter HIF mRNA levels. Furthermore, we demonstrate that MBZ can reduce HIF transcriptional activity in hypoxic tumor regions using preclinical xenograft and PDX mouse models of breast cancer. Taken together, our results suggest that MBZ treatment has additional therapeutic efficacy in hypoxic tumor regions. The results warrant further consideration of MBZ as a therapeutic strategy for treating solid tumors.

## 2. Materials and Methods

### 2.1. Cell Lines and Cell Culture

Mycoplasma-free breast cancer cell lines MDA-MB-231 and MCF-7 were purchased from the American Type Culture Collection (ATCC) and maintained in DMEM (Corning) supplemented with 10% FBS (Corning) and 1% penicillin/streptomycin (P/S) (Invitrogen). SUM159 cells were kindly provided by the Sukumar Lab and cultured in Ham’s F12 medium supplemented with 5% FBS, 1% P/S, and 5% insulin/hydrocortisone. Cells were maintained in a humidified environment at 37 °C and 5% CO_2_. Hypoxia fate-mapping MDA-MB-231 cells were developed and maintained as previously described [24].

### 2.2. CLUE Query (Clue.io from the BROAD Institute)

A connectivity map analysis used two reference datasets, (1) Touchstone v1, which has over 8000 well-annotated genetic and small molecule drug perturbagens profiled in a core set of nine cell lines, and (2) the Discover v1 dataset with over 15,000 unannotated small molecular perturbagens tested in a variety of cell lines. The combined databases created the L1000-based compendium CMAP-L1000v1 used to develop the CMap query. We used the top 100 genes that were found using RNA sequencing to be the most differentially downregulated (see Supplementary Table S1) in both MDA-MB-231 and SUM159 cells upon MBZ treatment to perform a CMAP query. Each PCL (perturbagen class) has an assigned mechanism of action (MOA) class with likely targets. Targets with shared MOAs are placed within the same class. The CMAP query provides a connectivity score for each perturbagen. The higher the score, the more likely MBZ shares a MOA with a given perturbagen.

A separate analysis was conducted using the Touchstone v1 dataset, which included mebendazole as a perturbagen. The expression profile of a cell line treated with mebendazole (MBZ) could be compared to other perturbagens used to treat the same cell line. This query compared the transcriptomic profile of the specified perturbagen, MBZ, and identified a similar profile across six cancer cell lines against other compounds that have a similar mechanism of action. In this case, a score of 1 to 100 is provided, with 100 being a perfect match to MBZ.

### 2.3. RNA Sample Preparation and Sequencing

MDA-MB-231, MCF-7, and SUM159 cells were collected, and total RNA was extracted. The samples had a RIN value > 9.0. From 1 μg of total RNA, mRNA was purified with poly-T oligo-attached magnetic beads. Next, double-stranded cDNA was synthesized, the cDNA fragments were adenylated, and sequencing adaptors were incorporated. Each fragment of 150–200 bp in length was purified, followed by PCR. Novogene sequenced the samples on a NovaSeq 6000 system with a 150-bp paired-end run. The reads were mapped to the Homo sapiens genome (GRCh38/hg38) using STAR (v2.5) with the parameter mismatch = 2. Quantification was carried out using HTSeq (v0.6.1) software with the parameter-m union. Differentially expressed genes were identified using EdgeR (v3.16.5) with padj < 0.005 and [log2(FoldChange)] > 1. FASTQ files and read counts have been uploaded to GEO under accession number GSE222702.

### 2.4. Western Blotting

Lysates from MDA-MB-231, SUM159, and MCF-7 cells were prepared in a buffer containing 150 mM NaCl, 1% IGEPAL CA-630, 50 mM Tris-HCL, pH 8.0, and protease and phosphatase inhibitors for 10 min on ice, and centrifuged for 10 min at 12,000 rpm at 4 °C. The supernatant was collected and fractionated on a 10% sodium dodecyl sulfate-polyacrylamide gel electrophoresis system (SDS-PAGE). The gel was then transferred to a nitrocellulose membrane using a Trans-blot Turbo (BioRad, Hercules, CA, USA). Each membrane was blocked in 5% milk (% *w*/*v*) prepared in Tris-buffered saline containing 0.1% Tween-20 (TBS-T). The membrane was incubated overnight in primary antibodies (HIF-1α (610959, BD Biosciences, San Jose, CA, USA), HIF-2α (SC-46691, Santa Cruz Biotechnology, Dallas, TX, USA), HIF-1β/ARNT (D28F3, Cell Signaling Technology, Danvers, MA, USA), NDRG1 (A18057, ABclonal Science, Woburn, MA, USA), and CA9 (SC-365900, Santa Cruz Biotechnology)) at a 1:1000 dilution at 4 °C. After three washes in TBS-T, membranes were incubated in HRP-conjugated secondary antibodies (Cell Signaling Technology), followed by three additional washes in TBS-T. HRP-conjugated Beta Actin Monoclonal antibody (HRP-60008, Proteintech, Rosemont, IL, USA) was used at a 1:5000 dilution to detect actin as the loading control. The chemiluminescence signal was detected using an AZURE C300 (Azure^TM^ Biosystems, Dublin, CA, USA) using ECL as the chemiluminescent substrate (Perkin Elmer, Waltham, MA, USA).

### 2.5. Reverse Transcription and Quantitative PCR

Total RNA was extracted using TRIzol (Invitrogen, Waltham, MA, USA) followed by cDNA synthesis (GoScript^TM^, Promega, Madison, WI, USA). qPCR analysis was conducted on a CFX96 Real-Time PCR detection system (Bio-Rad) using SYBR Green qPCR master mix (Bio-Rad and ABclonal Science). The expression of each target mRNA relative to 18S rRNA control was calculated as 2^−Δ(ΔCt)^, ΔCt = Ct (target mRNA) − Ct (18S rRNA), and Δ(ΔCt) = ΔCt (treatment) − ΔCt (control). Primer sequences are as follows: BNIP3 FW: 5′-CTTCCATCTCTGCTGCTCTC-3′; BNIP3 RV: 5′-GTAATCCACTAACGAACCAAGTC-3′; CA9 FW: 5′-GGATCTACCTACTGTTGAGGCT-3′; CA9 RV: 5′-CATAGCGCCAATGACTCTGGT-3′; EPO FW: 5′-GCCCTACGTGCTGTCTCACAC-3′; EPO RV: 5′-CCTTGATGACAATCTCAGCGC-3′; NDRG1 FW: 5′-CCAACAAAGACCACTCTCCTC-3′; NDRG1 RV: 5′-CCATGCCCTGCACGAAGTA-3′; 18S FW: 5′-GAGGATGAGGTGGAACGTGT-3′; and 18S RV: AGAAGTGACGCAGCCCTCTA-3′.

### 2.6. Firefly Luminescence Assay

MDA-MB-231 cells were seeded overnight in 24-well plates and co-transfected with 0.4 μg of the indicated vectors, 0.05 μg of psVmRL *Renilla* luciferase vector, and 0.05 μg of pcDNA3-EGFP per well using PolyJet in vitro DNA Transfection Reagent (SignaGen Laboratories). The internal control was a *Renilla* luciferase vector. After 16 h, the medium was changed, and the transfected cells were exposed to 20% or 1% O_2_ and treated with 1 μM MBZ or DMSO vehicle control and analyzed for luciferase activity using a Dual-Luciferase Reporter Assay System per the manufacturer’s instruction (Promega). Luminescence was measured using a Cytation 5 (BioTek Instruments, Winooski, VT, USA).

### 2.7. Preclinical Experiments In Vivo

All animal research complied with relevant ethical regulations within protocols approved by the Johns Hopkins University Animal Care and Use Committee. Female 5- to 7-week-old NOD-SCID Gamma (NSG) mice were anesthetized with intraperitoneal injections of 100 mg/kg Ketamine and 16 mg/kg Xylazine prior to cell or PDX implantation.

#### 2.7.1. MDA-MB-231 Xenograft Mouse Model

MDA-MB-231 hypoxia fate-mapping cells (2 × 10^6^) were injected into the top mammary fat pad closest to the second nipple. At the end of the experiment (36 days), tumors were excised, formalin-fixed (Sigma-Aldrich, St. Louis, MO, USA) overnight, saturated in 30% sucrose (Sigma-Aldrich) at 4 °C overnight, and frozen in OCT media (Fisher Scientific, Waltham, MA, USA). Tumors were sectioned using a cryotome CM11000 (Leica, Wetzlar, Germany) and mounted onto Superfrost Plus microscope slides. The slides were imaged to detect DsRed and GFP expression using a Cytation 5 (BioTek Instruments). Quantification was carried out using ImageJ by calculating the area of each signal in the respective channels (RFP, GFP, RFP + GFP). After thresholding and binarization, the measure plugin was used to obtain a % score for GFP compared to the total tissue area.

#### 2.7.2. Breast Cancer Patient-Derived Xenograft (PDX)

The HCI-001 PDX was developed by the Welm lab [25]. HCI-001 PDX tumor fragments were maintained and passaged in NOD-SCID Gamma (NSG) mice. Tumors were collected and serially re-implanted into new mice in ~1 mm size fragments or cryopreserved and passages were tracked. For experiments, PDX fragments were dissociated by incubating in 2 mg/mL collagenase for 1 h at 37 °C, and DNase I (0.4 U/mL) (Sigma-Aldrich) was added for 5 min at room temperature. The cell suspension was centrifuged, resuspended in fresh media, and strained through a 100 μm Nylon filter. Fragments (n = 5000) smaller than 100 μm were re-implanted into each recipient NSG mouse.

When the tumor size reached 150 mm^3^, mice were treated with MBZ. Once the tumor burden of the control group reached 1 cm^3^, mice were sacrificed, and tumor tissue was preserved for RNA extraction and paraffin embedment. For RNA extraction, small tumor fragments were mechanically dissociated in TRIzol (Invitrogen). RNA extraction was performed with phenol:chloroform:isoamyl alcohol (25:24:1) (Sigma), followed by precipitation with isopropanol (Sigma). RNA pellets were washed with ethanol (70%), air-dried, and resuspended in RNAse/DNAse-free water.

### 2.8. Fluorescent Staining of Formalin-Fixed and Paraffin-Embedded (FFPE) Tissue Sections

Formalin-fixed and paraffin-embedded tissue sections of PDX tumors were subjected to antigen retrieval using citrate buffer (pH 6.2) at 60 °C for 20 min post-deparaffinization with xylenes and serial hydration. Hydrogen peroxide (3% *v*/*v*) diluted in methanol was using to quench intrinsic peroxidase activity. PBS-T was used for washes and samples were blocked with blocking buffer (0.05% Tween, 1% Casein, 2% BSA and 5% Goat serum, in PBS) for 1 h followed by a 4 °C overnight primary antibody incubation with a CA9 antibody (#sc-365900, SantaCruz, Santa Cruz, CA, USA) at 1:100 dilution. Samples were washed with PBS-T on the following day, and incubated with HRP goat anti-mouse (#20401, Biotium, Fremont, CA, USA) secondary antibody at 1:200 dilution prepared in blocking buffer for 1 h. The slides were then subjected to signal amplification using a tyramide amplification kit (#33012, Biotium). DAPI staining was performed for 15 min at room temperature. Slides were mounted with anti-fade solution and imaged on a Cytation 5 device (BioTek Instruments). The digital score was obtained by calculating the signal for CA9 and DAPI using the threshold plugin on ImageJ and taking their ratio. See Supplementary S2 for an example of the digital scoring and staining for each PDX tumor.

### 2.9. Statistical Analysis

GraphPad Prism 9 was used for statistical analysis, and statistical tests appropriate for each experimental setup were performed. Data are presented as mean ± SEM. A comparison of two-variable data was performed with an unmatched two-way ANOVA using Bonferroni multi-comparison testing. Significance levels are reported as indicated in the figure legends.

## 3. Results

### 3.1. Connectivity Map Analysis of Transcriptional Profile Changes Caused by MBZ Treatment Uncovers a Novel Link to Hypoxia-Inducible Factor (HIF) Inhibitors

**Figure 1:**
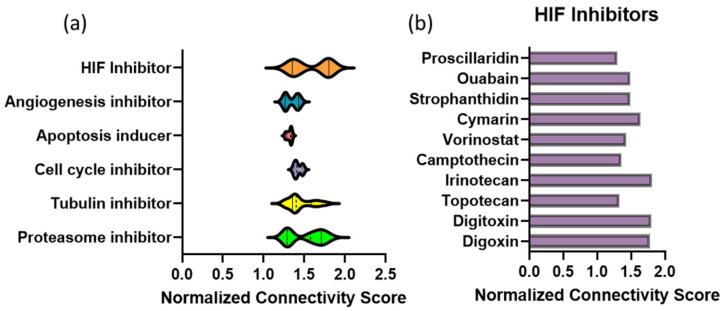
Connectivity map analysis of transcriptional profile changes caused by MBZ treatment uncovers a novel link to hypoxia-inducible factor (HIF) inhibitors. (**a**) CLUE query from the Broad Institute. Normalized connectivity scores of inhibitors and inducers based on the top 100 differentially expressed genes decreased in both MDA-MB-231 and SUM159 cells treated with MBZ in vitro based on RNA sequencing data. Violin plot of highest connectivity score for the classes of perturbations indicated. Proteasome inhibitors (n = 14), cell cycle inhibitors (n = 3), apoptosis inducers (n = 5), angiogenesis inhibitors (n = 4), HIF-1α inhibitors (n = 6), and tubulin inhibitors (n = 17). (**b**) Bar graph of specific normalized connectivity scores for HIF-1α inhibitors shown in (**a**).

Utilizing our previous RNA sequencing data from two human TNBC cells lines (MDA-MB-231 and SUM159, (GSE190845)) treated with MBZ, we focused on the 100 differentially expressed genes (Table S1) that were downregulated with MBZ treatment. We input the downregulated gene set into the Broad Institute’s clue.io database to generate a connectivity map based on two large datasets (Touchstone v1 and Discover v1) of perturbagens and their transcriptomic profiles in a multitude of cell lines classified by mechanisms of action. Through our query, we uncovered the top classes of perturbagens that also cause a reduction in the top 100 differently expressed genes (Table S1). Unsurprisingly, perturbations with known mechanisms of action for MBZ treatment [2] had high connectivity scores and included tubulin inhibitors, cell cycle inhibitors, apoptosis inducers, angiogenesis inhibitors, and proteasome inhibitors (Figure 1a). Interestingly, well-known HIF inhibitors [26] had connectivity scores within the same range as tubulin inhibitors, the most well-accepted mechanism of action for MBZ (Figure 1a,b).

We performed a second query using the Touchstone v1 dataset, which included MBZ as a perturbagen. Changes in gene expression caused by MBZ treatment were compared for similarity to all perturbational signatures in the following six cell lines: melanoma (A375 cells), breast cancer (MCF-7 cells), lung cancer (A549 cells), prostate cancer (PC3 cells), colon adenocarcinoma (HT29 cells), and an immortalized kidney cell line (HA1E) (Figure S1). A score of 1 to 100 is provided, with 100 being a perfect match to the transcriptional profile of MBZ treatment. As expected, benzimidazoles such as nocodazole, flubendazole, albendazole, oxibendazole, parbendazole, and fenbendazole had scores of greater than 95 in five out of the six cell lines queried. As our previous analysis suggested, HIF inhibitors [26], such as proscillaridin, ouabain, and digoxin, had scores greater than 85 in all six cell lines that were queried (Figure S1). Taken together, the data suggest that MBZ treatment and HIF inhibition cause similar transcriptional profile changes in cancer cell lines.

### 3.2. Mebendazole Decreases the Transcriptional Activity of HIFs

**Figure 2:**
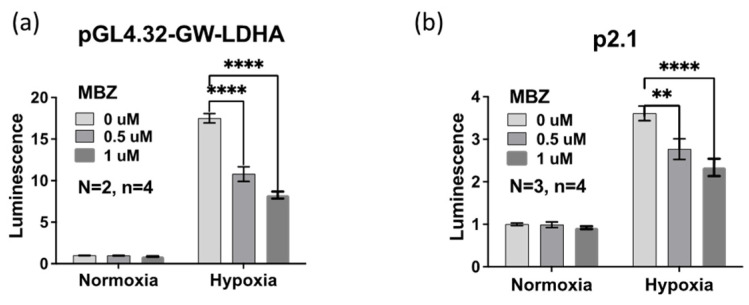
Mebendazole decreases the transcriptional activity of HIFs. (**a**,**b**) MDA-MB-231 cells were transfected with either the pGL4.32-GW-LDHA reporter plasmid (**a**) or the p2.1 reporter plasmid (**b**). The cells were exposed to 20% O_2_ (normoxic) or 1% O_2_ (hypoxic) conditions followed by 48 h of treatment with MBZ (0.5 μM and 1 μM) or DMSO as vehicle control. Firefly fluorescence was normalized to Renilla luciferase luminescence reporter assay. Mean ± SEM; n = 2 independent experiments with n = 4 technical replicates. *p*-values refer to one-way ANOVA test ** < 0.01 or **** < 0.0001.

To determine whether the similarity in gene signatures following treatment with MBZ or HIF inhibitors was correlative or causative, we tested the transcriptional activity of HIFs using a reporter assay. We transfected MDA-MB-231 cells with two HIF-dependent reporter vectors: the pGL4.32-GW-LDHA plasmid containing a human lactate dehydrogenase A (LDHA) HIF-promoter binding site or the p2.1 plasmid containing a human enolase HIF-promoter binding sequence. Both HIF-binding sequences were located upstream of a minimal promoter, followed by a firefly luciferase coding sequence. The psVmRL Renilla luciferase vector, which constitutively expresses Renilla luciferase, was used as a control reporter for normalization purposes [27]. Transfected MDA-MB-231 cells were treated with MBZ or vehicle control DMSO for 48 h under 20% or 1% O_2_. In both dual-luciferase assays, pGL4.32-GW-LDHA (Figure 2a) and p2.1 (Figure 2b), MBZ treatment lowered HIF transcriptional activity under hypoxia in a dose-dependent manner as measured by normalized luciferase expression. The results suggest that MBZ directly represses the hypoxia-induced HIF transcriptional response.

### 3.3. Mebendazole Decreases HIF-Inducible Gene Transcription under Hypoxic Conditions

**Figure 3:**
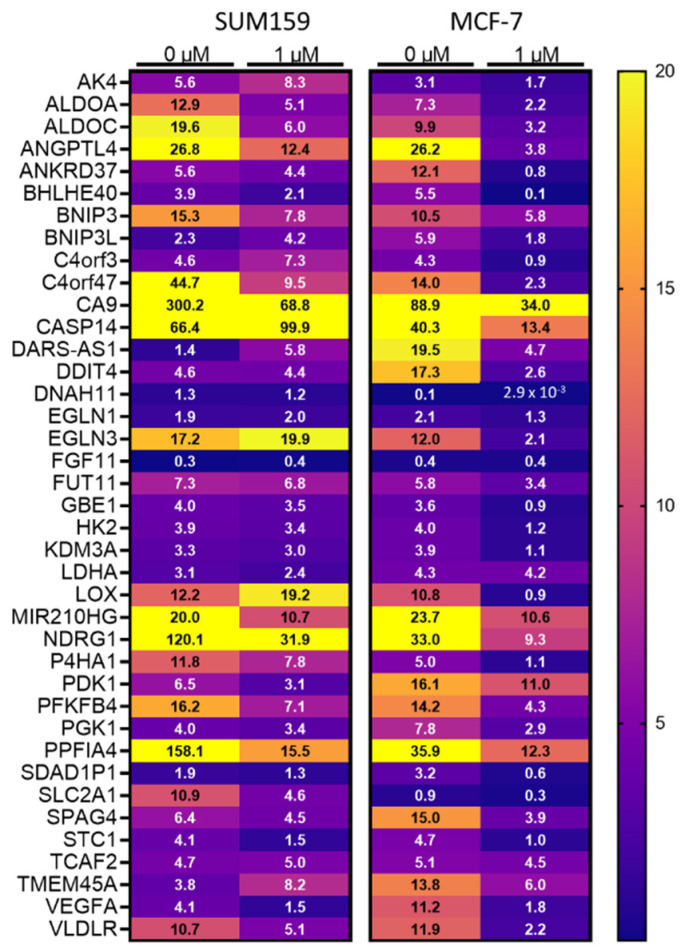
Mebendazole decreases HIF-dependent gene transcription under hypoxic conditions. Fold change in gene expression for each gene in the 42-gene hypoxia signature in SUM159 (**left**) or MCF-7 (**right**) cells treated with DMSO or 1 μM MBZ under 1% O_2_ versus 20% O_2_ conditions.

To determine whether MBZ inhibits HIF-dependent gene transcription under hypoxia, we performed RNA sequencing of SUM159, MDA-MB-231, and MCF-7 cells exposed to 20% O_2_ or 1% O_2_ conditions in the presence or absence of 1 μM MBZ or DMSO as vehicle control. We compared the expression of a previously published 42 breast cancer-specific hypoxia-inducible and HIF-regulated gene set [28] (Figure 3 and Figure S2). Our data indicated that MBZ caused a decrease in the overall expression of the hypoxic gene signature across all breast cancer cell lines, confirming that MBZ decreases HIF transcriptional activity.

### 3.4. Mebendazole Decreases HIF-Inducible Gene Products in a Dose-Dependent Manner

**Figure 4:**
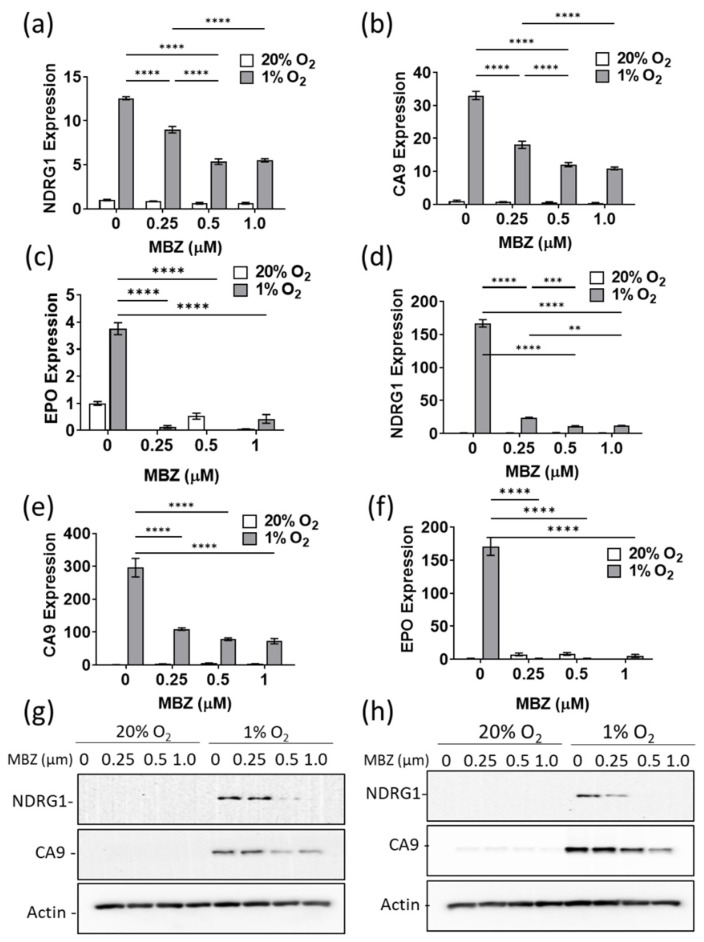
Mebendazole decreases HIF-inducible gene products in a dose-dependent manner. (**a**–**f**) Relative expression of NDRG1, CA9, and EPO measured by real-time-qPCRs of cDNA in MDA-MB-231 (**a**–**c**) and SUM159 (**d**–**f**) cells treated for 48 h with MBZ (0.25 μM, 0.5 μM, and 1 μM) or DMSO (vehicle control) exposed to 20% O_2_ or 1% O_2_. Mean ± SEM; n = 3 independent experiments and n = 3 technical replicates. *p*-values refer to one-way ANOVA test ** < 0.01,*** < 0.001,**** < 0.0001. (**g**,**h**) NDRG1 and CA9 protein expression were analyzed with an immunoblot assay using lysate extracted from MDA-MB-231 (**g**) and SUM159 (**h**) cell lines treated with increasing doses of MBZ as indicated. See Supplementary S1 for uncropped blots for (**g**,**h**).

To confirm the results of our RNA sequencing analysis, we measured the mRNA expression of four genes containing known hypoxia response elements (HRE): NDRG1, CA9, EPO, and BNIP3 in MDA-MB-231, SUM159, and MCF-7 cells (Figure 4a–f and Figure S3a–e) using real-time PCR. Hypoxia induces CA9 (carbonic anhydrase 9) in a HIF-1-dependent manner, EPO (erythropoietin) in a HIF-2-dependent manner and BNIP3 (BCL2 Interacting Protein 3) and NDRG1 (N-myc downstream regulated 1) in a HIF-1 and HIF-2- dependent manner. The induction of NDRG1, CA9, EPO1, and BNIP3 mRNA expression under hypoxic conditions was abrogated in a dose-dependent manner by MBZ treatment (Figure 4a–f and Figure S3a–e). Likewise, in all three cell lines, NDRG1 and CA9 protein expression were abrogated in a dose-dependent manner under hypoxic conditions (Figure 4g,h and Figure S3f). Taken together, the results confirm that MBZ affects the ability of both HIF1 and HIF2 to transactivate target genes under hypoxic conditions.

### 3.5. Mebendazole Decreases the Induction of HIF-1α, HIF-2α, and HIF-1β under Hypoxic Conditions

**Figure 5:**
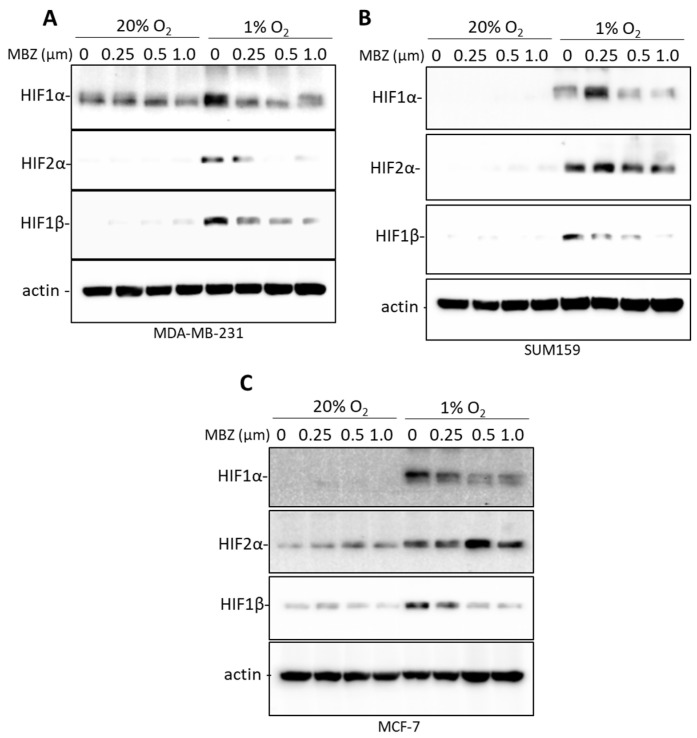
Mebendazole decreases the induction of HIF-1α, HIF-2α, and HIF-1β under hypoxic conditions. (**A**–**C**) HIF-1α, HIF-2α, and HIF-1β protein expression was analyzed with an immunoblot assay using lysate extracted from MDA-MB-231 (**A**), SUM159 (**B**), and MCF-7 (**C**) cells treated with increasing doses of MBZ as indicated. See Supplementary S1 for uncropped blots for this figure.

Hypoxia induces HIF-1 and HIF-2 complex formation by stabilizing the HIF-1α and HIF-2α subunits, each of which binds to the HIF-1β subunit to form the heterodimers HIF-1 and HIF-2, respectively [29]. Given that our results show that MBZ only abrogates HIF-dependent gene expression under 1% O_2_ but not 20% O_2_ conditions, we hypothesized that MBZ might directly affect the regulation of HIF expression under hypoxia. HIF-1α, HIF-2α, and, to our surprise, HIF-1β were induced in MCF-7, MDA-MB-231, and SUM159 cells exposed to 1% O_2_ for 48h (Figure 5A–C). Immunoblot assays further revealed that treatment of all three cell lines with MBZ for 48h inhibited HIF-1α, HIF-2α, and HIF-1β protein expression (Figure 5A–C). MBZ treatment of hypoxia-exposed cells caused a less than 10% reduction in HIF-1α, HIF-2α, and HIF-1β mRNA expression, except for HIF-2α expression in MCF-7 cells, which was reduced by 40%, and HIF-1β expression in SUM159 cells, which was reduced by 30% following MBZ treatment (Figure S4a–e).

### 3.6. Mebendazole Decreases HIF Transcriptional Activity In Vivo

**Figure 6:**
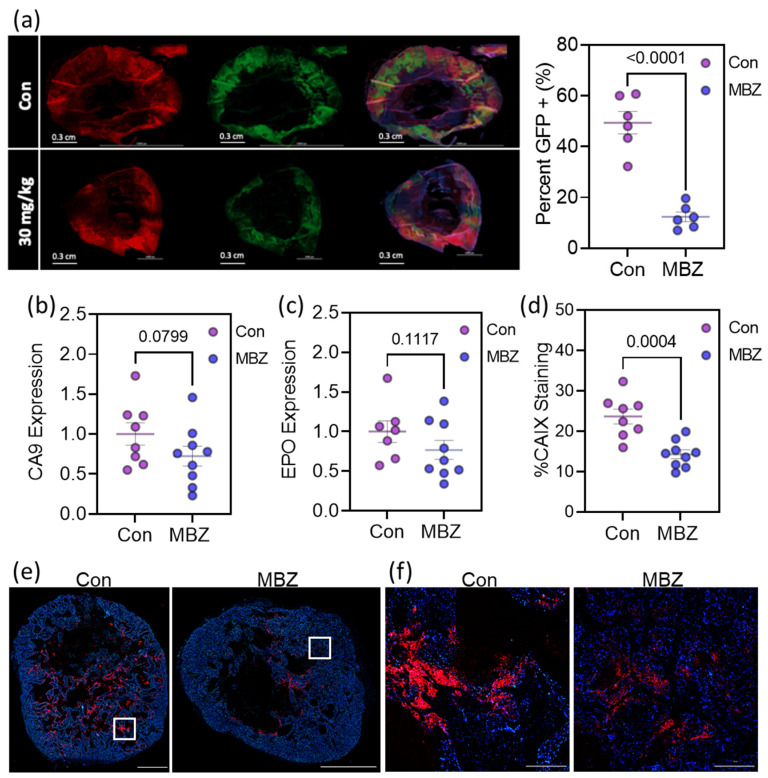
Mebendazole decreases HIF transcriptional activity in vivo. (**a**) Representative fluorescent image of full cross-sections of orthotopic mammary tumors derived from MDA-MB-231 hypoxia fate-mapping cells treated with sesame oil as vehicle control (n = 6) or 30 mg/kg of MBZ (n = 6) via oral gavage. The percentage of GFP expression in the tumors is graphed for each tumor on the right. (**b**–**d**) NSG mice were orthotopically implanted with a patient-derived xenograft model of triple-negative breast cancer, HCI-001. The mice were randomized and treated with sesame oil as a vehicle gavage (Con, n = 8) or 30 mg/kg MBZ in a sesame oil gavage (30 mg/kg; n = 9) four times per week. (**b**,**c**) Total RNA was isolated from tumor tissue and analyzed with RT-qPCR using primers specific for CA9 (**b**) or EPO (**c**) mRNA expression. The results were normalized to the mean value for tumors from vehicle-treated mice. *p*-values are indicated. (**d**–**f**) CA9 was immunofluorescently labeled and quantified for each tumor (**d**). Representative fluorescent images of sections of tumors stained for CA9 and quantified in (**d**) with scale bar = 3 mm (**e**). Enlarged insets shown in white are displayed in (**f**) with a scale bar = 300 µm. See Supplementary S2 for staining and imaging of each tumor as well as a methodology for quantification. Note: One sample measured for EPO mRNA expression had a result of not detected by real-time PCR, causing the control group to consist of seven replicates rather than 8.

Previously, our lab generated a fluorescently tagged MDA-MB-231 cell line that fate-maps cells that have been exposed to intratumoral hypoxia, resulting in a color switch from DsRed to GFP expression [24]. We orthotopically implanted NSG mice with these fluorescently tagged MDA-MB-231 cells and treated the mice with 30 mg/kg MBZ (oral gavage; n = 6) or vehicle control (sesame oil; n = 6) for 36 days. We isolated the primary tumors at the endpoint of the experiment and cryo-sectioned each tumor in order to quantify the percentage of GFP-positive cells. MBZ treatment resulted in a significant decrease in cells exposed to hypoxia (GFP+) within the primary tumor (Figure 6a) with a 27% decrease in tumor size, which was not statistically significant (not shown).

As a second model, we orthotopically implanted a patient-derived xenograft model of triple-negative breast cancer, HCI-001, in NSG mice. After 8 weeks, the primary tumors reached an average volume of ~150 mm^3^. The mice were randomized into two treatment groups with either sesame oil by oral gavage (Con; n = 8) or 30 mg/kg MBZ in a sesame oil gavage (30 mg/kg; n = 9) four times per week. After two weeks of treatment, a portion of the tumor was collected to extract RNA, and a second portion was collected for immunofluorescence staining. HIF-1-dependent CA9 mRNA expression and HIF-2-dependent EPO mRNA expression were both decreased in the tumors collected from MBZ-treated mice (Figure 6b,c). Likewise, immunofluorescent staining, imaging, and quantification of the expression of the HIF-1 inducible gene, CA9, showed a dramatic reduction in tumors from MBZ-treated mice (Figure 6d–f). While MBZ did not reduce tumor growth, we observed a two-fold reduction in lung metastasis in mice treated with 30 mg/kg MBZ compared to those given the vehicle control, as previously reported [16].

## 4. Discussion

Hypoxia induction within solid tumors causes disease progression and remains a key pathway to target when developing cancer therapeutics [30,31]. HIF-1α was identified in 1991, followed by HIF-2. Both are required for regulating over one-thousand genes under hypoxic conditions [31]. HIF-regulated genes are involved in pathways such as angiogenesis, invasion, metabolism, and metastasis [20]. In cancer, the hypoxic-induced HIF expression is linked to resistance to radiation and chemotherapies, and lower overall survival partly due to promoting metastasis [22,32]. Therefore, drugs that offer a chemotherapeutic effect and have the added benefit of killing hypoxic cells would be vital to preventing cancer recurrence.

We have recently been studying the preclinical efficacy of MBZ, a drug used to treat parasitic infections [1], as a prevention or treatment strategy for breast cancer metastasis [15]. MBZ significantly reduced breast metastasis to the lung and abolished metastasis to the liver in three preclinical breast cancer metastasis models [15]. Likewise, MBZ has been shown to have preclinical efficacy in treating pancreatic [3], lung [4,5], thyroid [6], breast [7], colorectal [8], and brain cancer [9,10,11]. In addition, a phase I clinical trial treating adults with high-grade glioma demonstrated that MBZ is safe and tolerable [33]. We and others showed that MBZ decreased cell proliferation through previously described MOA, including tubulin disruption, G2/M cell cycle arrest, and apoptosis [6,15]. We questioned whether there were additional MOAs that promoted the anticancer effect of MBZ.

Our initial RNA sequencing data for estrogen receptor-negative (MDA-MB-231, SUM159) and estrogen receptor-positive (MCF-7) cell lines treated with MBZ showed downregulation of key genes known to be transcriptionally activated by HIFs under hypoxic conditions. RNA sequencing of cells exposed to 20% or 1% O_2_ conditions in the presence or absence of MBZ demonstrated that MBZ prevents the upregulation of a 42-gene hypoxia signature specific to breast cancer cells when exposed to hypoxia. Additionally, in all three cell lines, MBZ treatment prevented the induction of HIF-responsive genes and their subsequent protein expression and hypoxia-dependent transcriptional activity. Mice treated with 30 mg/kg MBZ had a reduction in HIF-dependent transcriptional activity in their tumors and a decrease in the population of viable cells exposed to hypoxia (GFP + cells). Furthermore, MBZ treatment resulted in decreased lung metastasis. This is in line with our previous findings using the hypoxia fate-mapping approach, where we demonstrate that cells that experience hypoxia in the primary tumor (GFP+) are more likely to contribute to lung metastasis, as compared to cells that were never exposed to hypoxia (DsRed+) [22]. Taken altogether, in addition to the previously recognized MOA that defines the therapeutic efficacy of MBZ, we find that it reduces the transcriptional activity of HIFs, whose gene products are well known to be required for metastasis [34].

It is well established that increased protein synthesis and decreased protein degradation lead to increased HIF-1α and HIF-2α protein levels in many cancers [35]. Additional experiments are required to determine whether MBZ decreases HIF expression under hypoxia via the PHD-VHL-proteasome pathway. For example, testing whether MG132, a proteasome inhibitor, prevents the MBZ-dependent decrease in HIF expression would provide additional mechanistic insight. On the other hand, MBZ could decrease the rates of HIFα protein synthesis or translation. Likewise, the lysosomal degradation of HIF-1α could occur [36].

In addition to regulating HIF-1α and HIF-2α, we find that HIF-1β is also decreased under hypoxic conditions in the breast cancer cell lines we assessed. However, the overall contribution of the decrease in HIF-1β levels caused by MBZ treatment to preventing an HIF-dependent transcriptional activity under hypoxia is also unknown. HIF-1β is often thought to be constitutively expressed [37], but its induction following hypoxia has been previously reported [38].

## 5. Conclusions

Our data warrant further elucidation of the exact mechanism by which MBZ affects HIF regulation. The ability of MBZ to reduce HIF-regulated gene expression in both estrogen receptor-positive (MCF-7) and estrogen receptor-negative (MDA_MB-231/SUM-159) cells adds to the versatility of MBZ [39] as an agent that reduces tumor growth and prevents metastatic disease. Patients with cancer are likely to benefit not only from the chemotherapeutic-like properties of MBZ treatment but from the prevention of the chemoresistant phenotype of hypoxic cancer cells.

## Data Availability

RNA sequencing data are available using GEO database accession number GSE222702.

## Supplementary Materials

The following supporting information can be downloaded at: https://www.mdpi.com/article/10.3390/cancers15041330/s1. Figure S1: The transcriptional profile following MBZ treatment is similar across six cancer cell lines and includes HIF inhibitors; Figure S2: Mebendazole decreases the hypoxia-induced expression of the 42-gene hypoxia breast cancer signature in MDA-MB-231 cells; Figure S3: Mebendazole reduces the expression of HIF-inducible gene products in a dose-dependent manner; Figure S4: Mebendazole does not significantly alter the mRNA expression of the HIF1, EPAS/HIF-2α, or ARNT/HIF-1β genes; Table S1: Top 100 differentially decreased genes following MBZ treatment in MDA-MB-231 and SUM159 cells used for the clue.io query; Supplementary S1: Uncropped immunoblots for Figure 4g,h, Figure 5, and Figure S3c; Supplementary S2: PDX tumors stained for CA9 and quantified using Image J as presented in Figure 6D.
