# Supplementary material for: Mebendazole Treatment Disrupts the Transcriptional Activity of Hypoxia-Inducible Factors 1 and 2 in Breast Cancer Cells
Source: Cancers (Basel). 2023 Feb 20;15(4):1330. doi: 10.3390/cancers15041330 (PMC9954103; doi:10.3390/cancers15041330)
Supplement: Supplementary file 1 [file cancers-15-01330-s001.zip › Supplemental Figures.pdf]

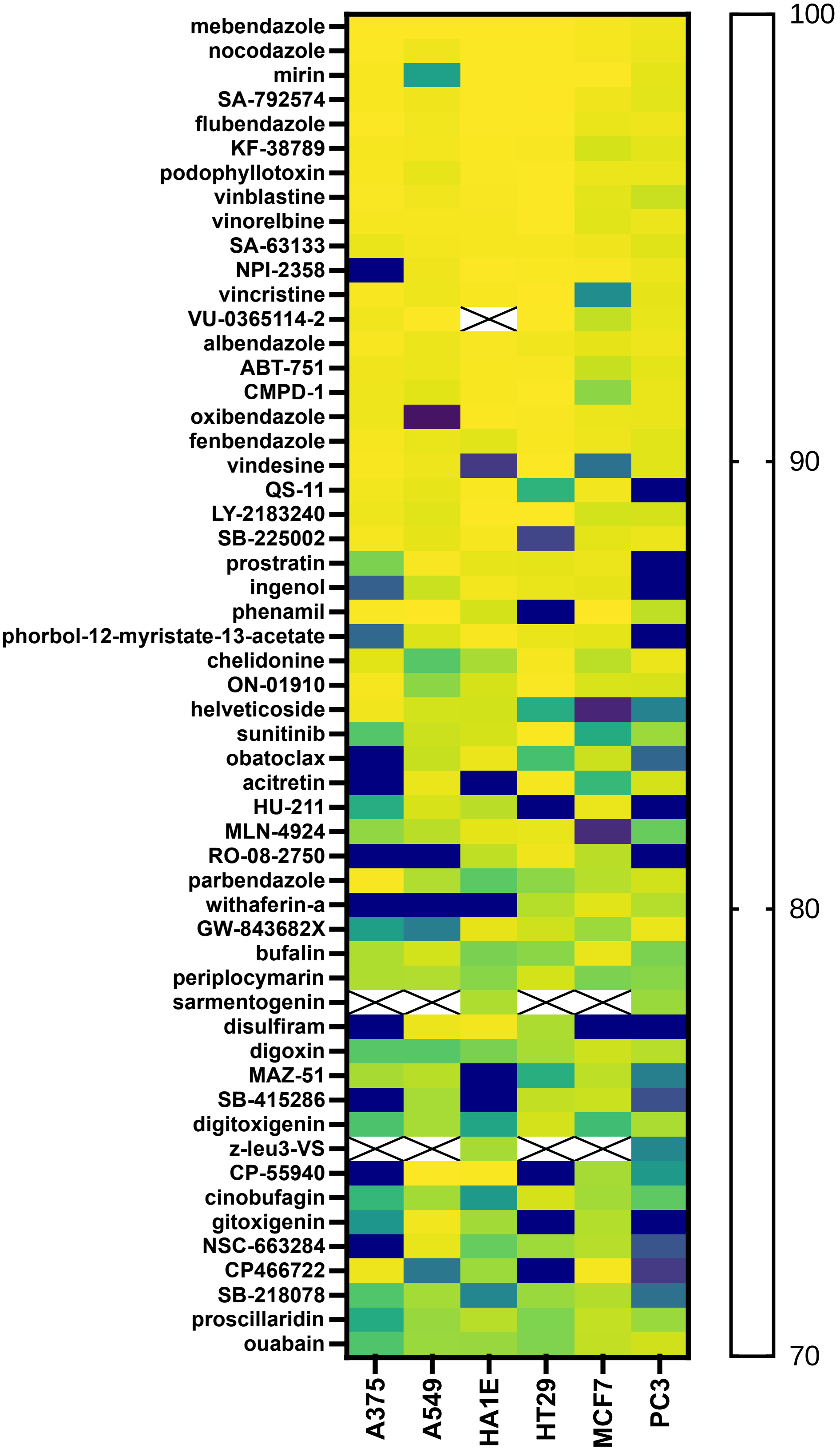

Figure S1. *The transcriptional profile following MBZ treatment is similar across six cancer cell lines and includes HIF-inhibitors.* Heatmap of well-annotated small molecular perturbations that result in a similar transcriptomic profile as MBZ treatment in a core set of 6 cancer cell lines using the *Touchstone v1 dataset.*

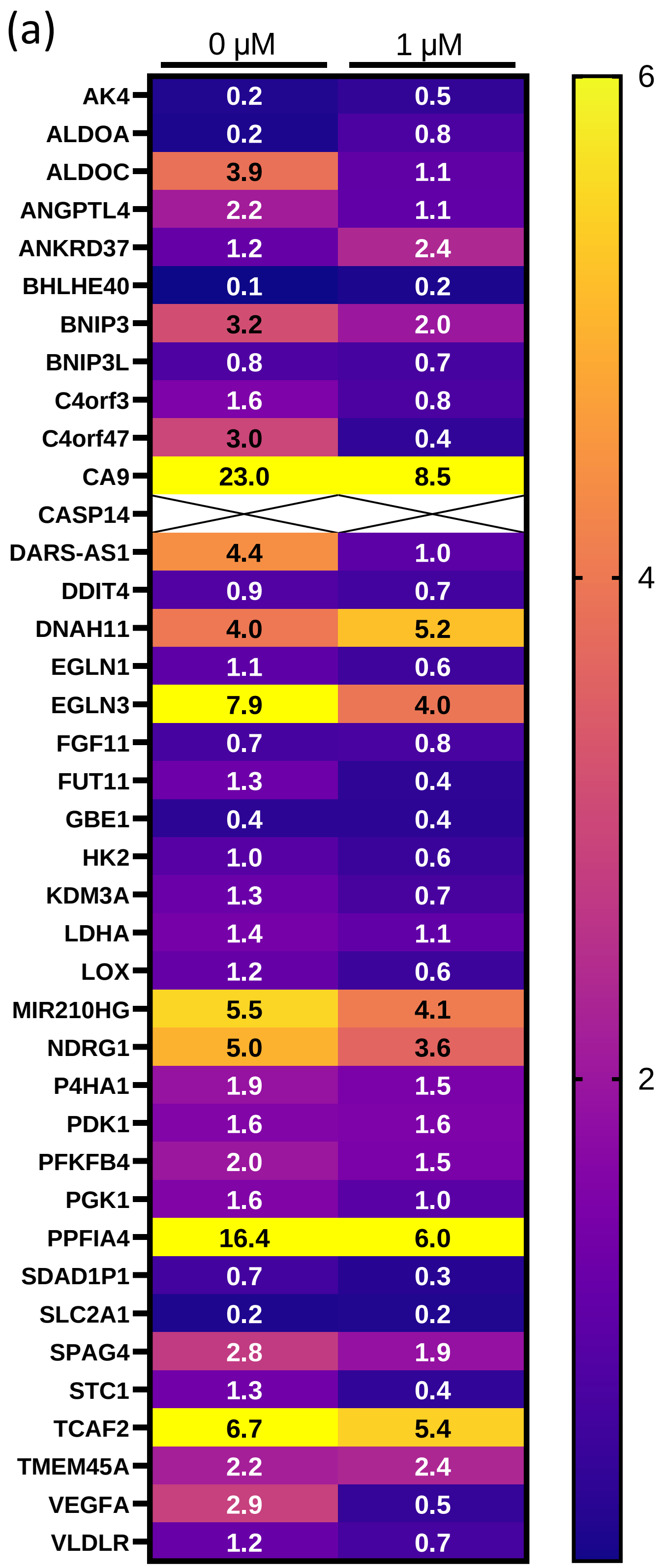

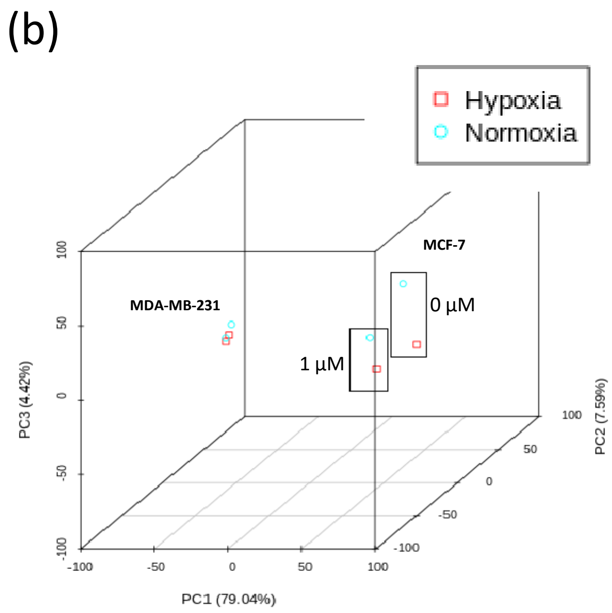

Figure S2. *Mebendazole decreases the hypoxia-induced expression of a 42-gene hypoxia breast cancer signature in MDA-MB-231 cells* (a) Heatmap of the fold change in gene expression values for the 42-gene hypoxia signature in MDA-MB-231 cells treated in the presence (1 µM) or absence (0 µM) of MBZ under 1% versus 20% O_2 conditions. (b) 3D PCA plot of MDA-MB-231 or MCF-7 gene expression in the presence (1 µM) or absence (0 µM) of MBZ and exposed to 1% or 20% O_2 conditions.

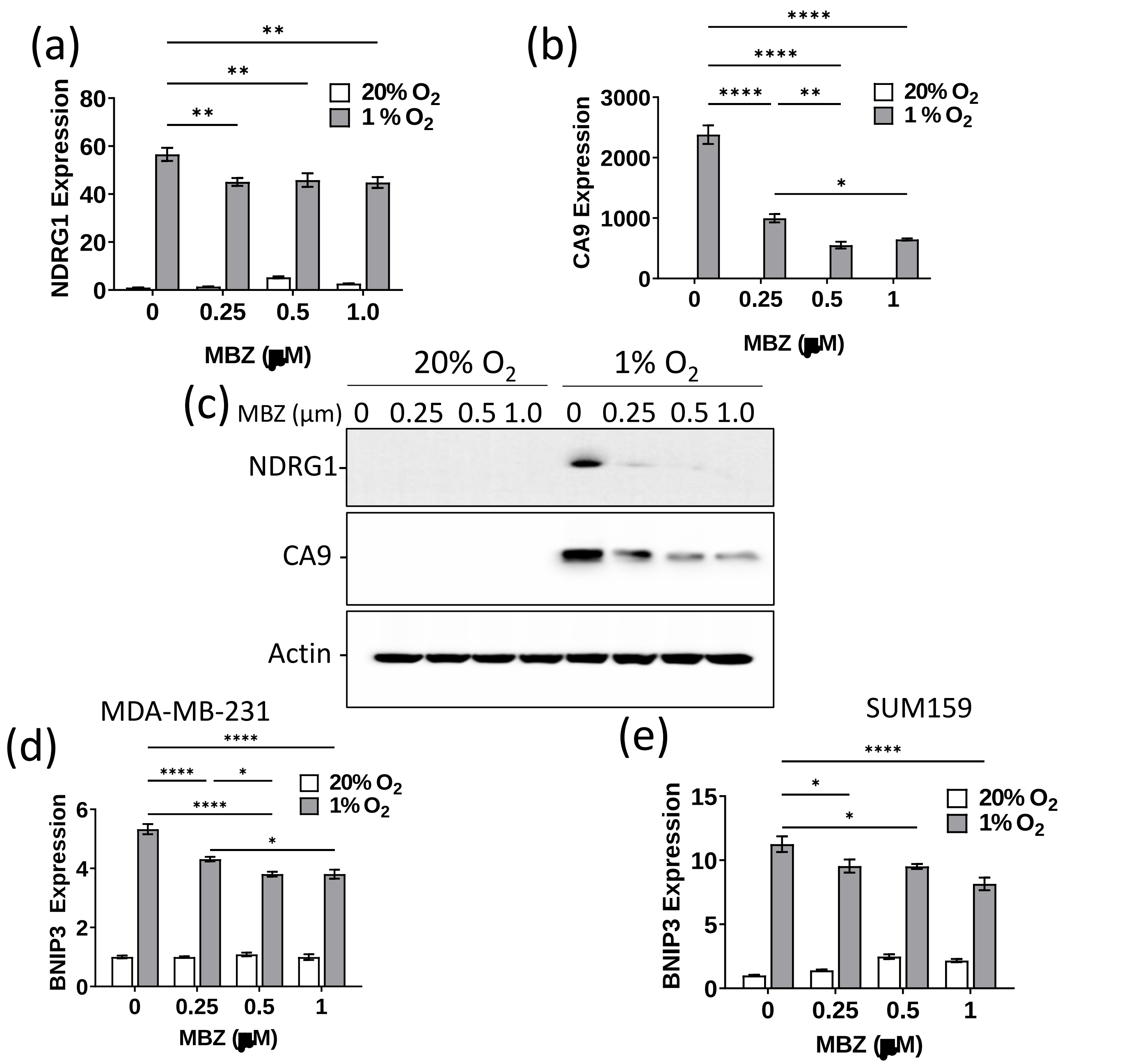

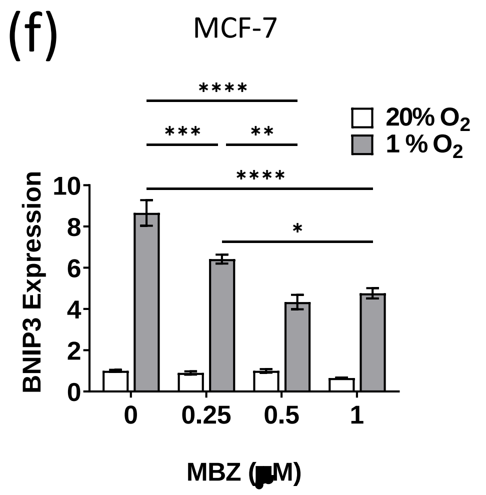

Figure S3. *Mebendazole reduces the expression of HIF-inducible gene products in a dose-dependent manner*. (a,b) Relative expression of NDRG1 and CA9 measured by real-time-qPCRs of cDNA generated from MCF-7 cells treated for 48h with MBZ (0.25 µM, 0.5 µM, and 1 µM) or DMSO (as a vehicle control) exposed to 20% O_2 or 1% O_2. Mean $\pm$ SEM; N = 3 independent experiments with n=3 technical replicates. P-values refer to one-way ANOVA test *<0.05, **<0.01,****<0.0001. (c) NDRG1 and CA9 protein expression analyzed by immunoblot assay using lysate extracted from MCF-7 cells treated with increasing doses of MBZ as indicated. See Appendix A for uncropped blots. (d-f) Relative expression of BNIP3 as measured by real-time-qPCRs of cDNA generated from MDA-MB-231 (d), SUM159 (e), or MCF-7 (f) treated as described in (a,b). Mean $\pm$ SEM; N = 3 independent experiments with n=3 technical replicates. P-values refer to one-way ANOVA test *<0.05, **<0.01, ***<0.001, ****<0.0001.

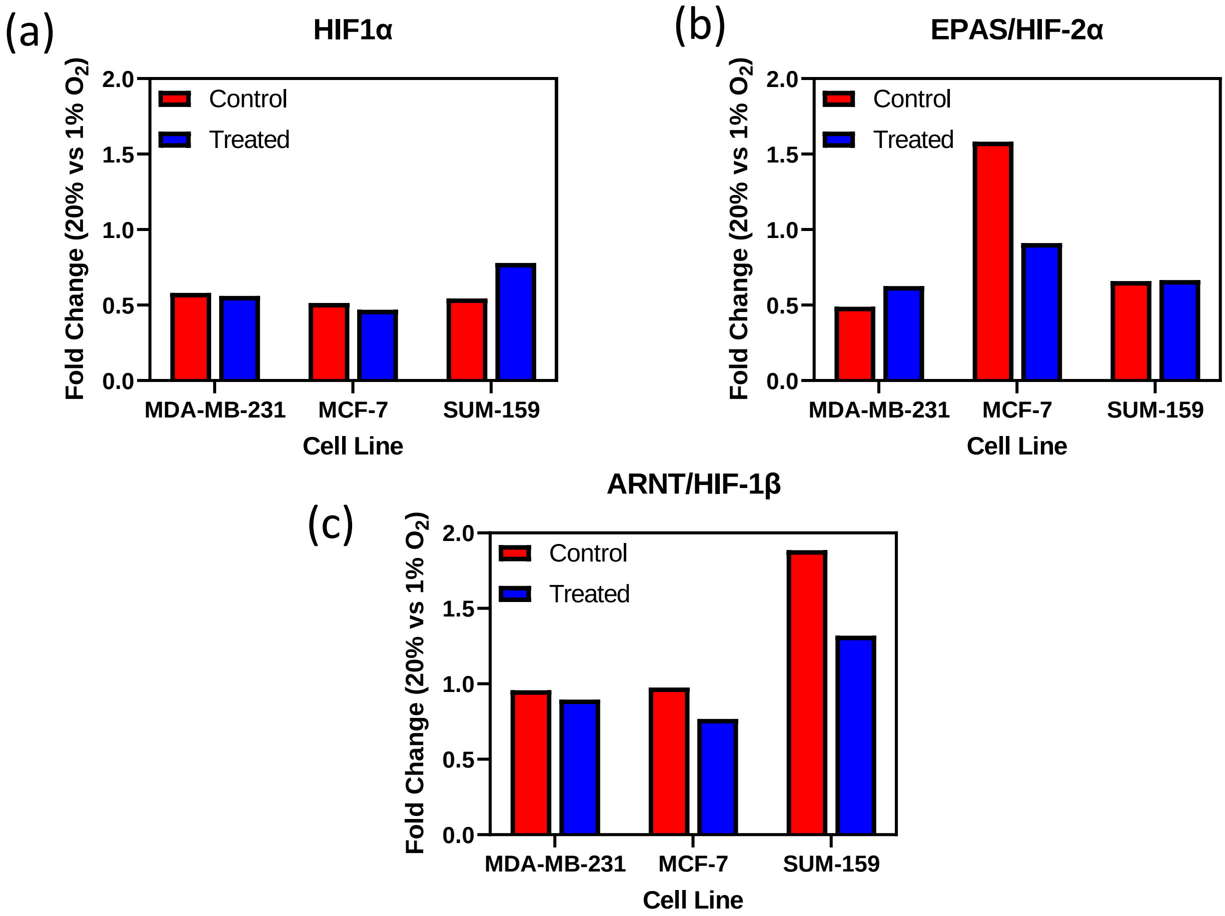

Figure S4. *Mebendazole does not significantly alter the mRNA expression of the HIF1, EPAS/HIF-2α, or ARNT/HIF-1*β *genes;.* The fold change of the normalized counts for the *HIF-1* (a) gene which encodes HIF-1α , *EPAS* (b) gene which encodes HIF-2α , or the *ARNT* (c) gene that encodes HIF-1β obtained by RNA sequencing data and quantified for MDA-MB-231, MCF-7, or SUM159 cells treated with DMSO as a vehicle control (control) or 1 µm of MBZ (treated).
