# Supplementary material for: Mebendazole Treatment Disrupts the Transcriptional Activity of Hypoxia-Inducible Factors 1 and 2 in Breast Cancer Cells
Source: Cancers (Basel). 2023 Feb 20;15(4):1330. doi: 10.3390/cancers15041330 (PMC9954103; doi:10.3390/cancers15041330)

Figure 3H: SUM159 uncropped

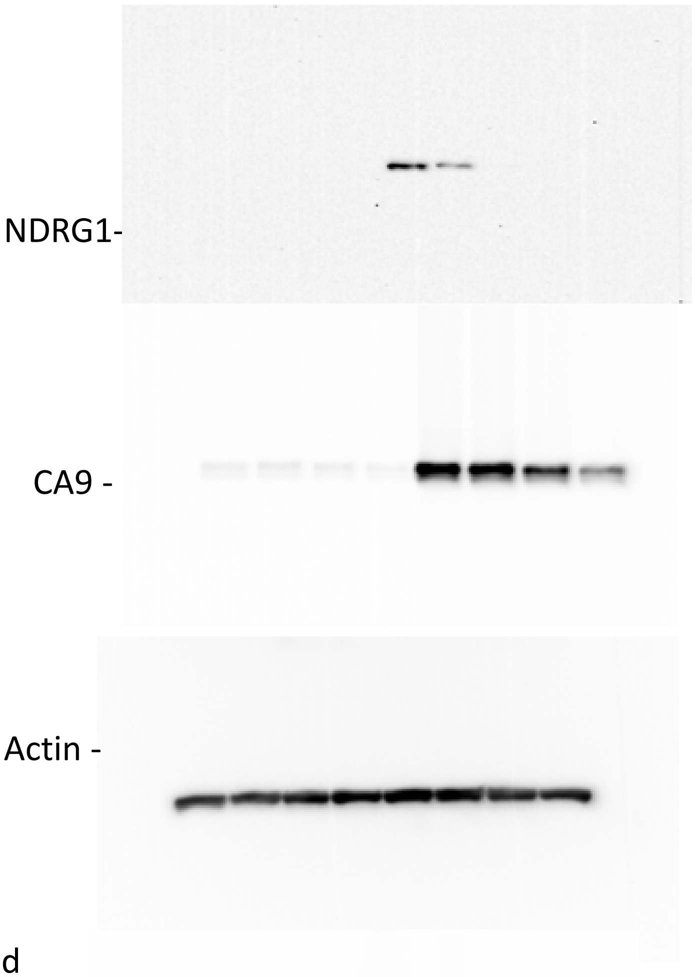


Figure 3D: MDA-MB-231 uncropped

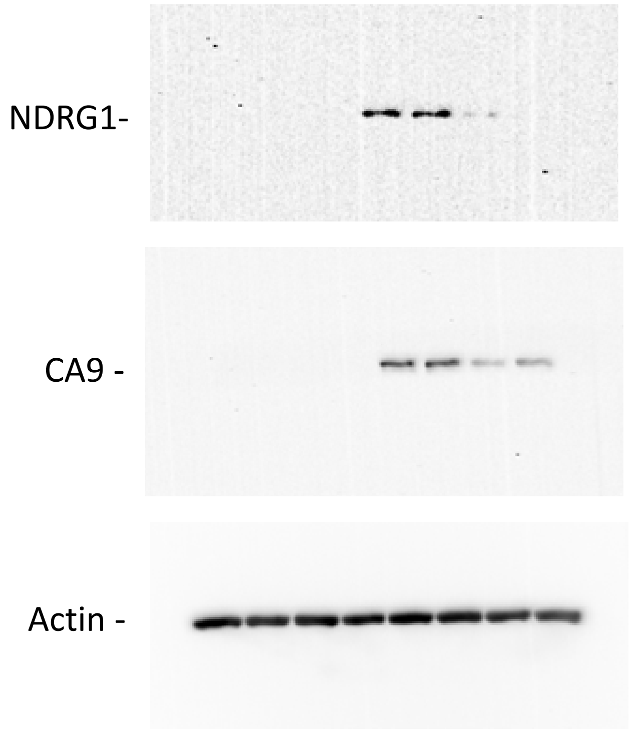


Supplemental 3D and 4C: MCF7 uncropped

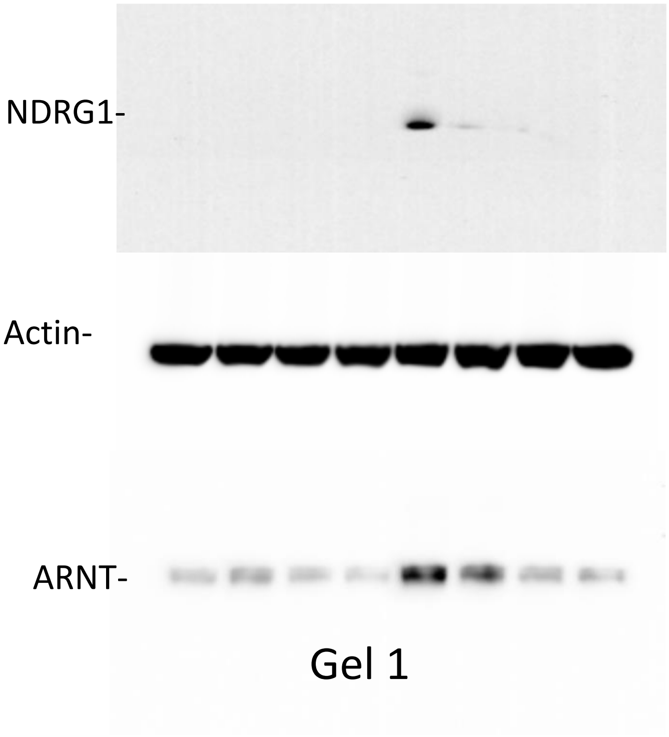


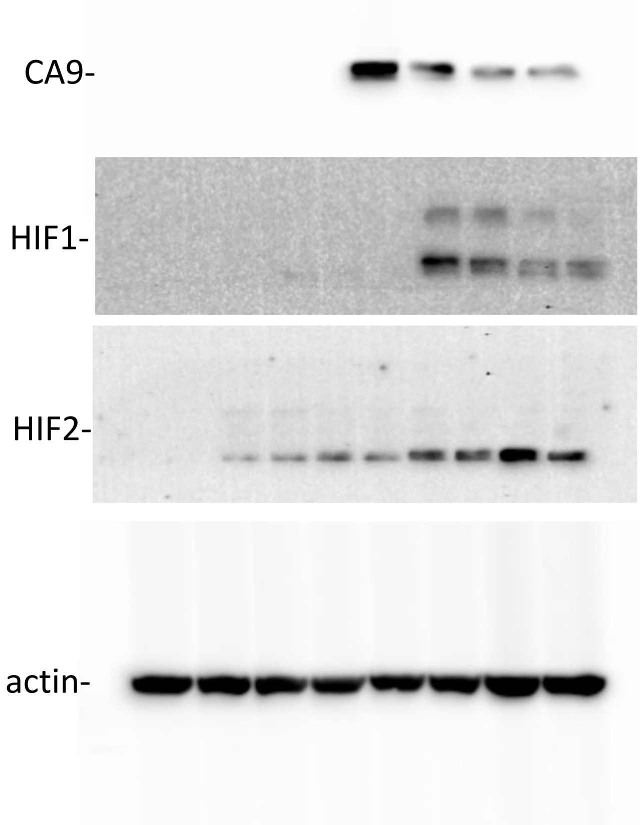

Figure 4A:  MDA-MB-231 uncropped

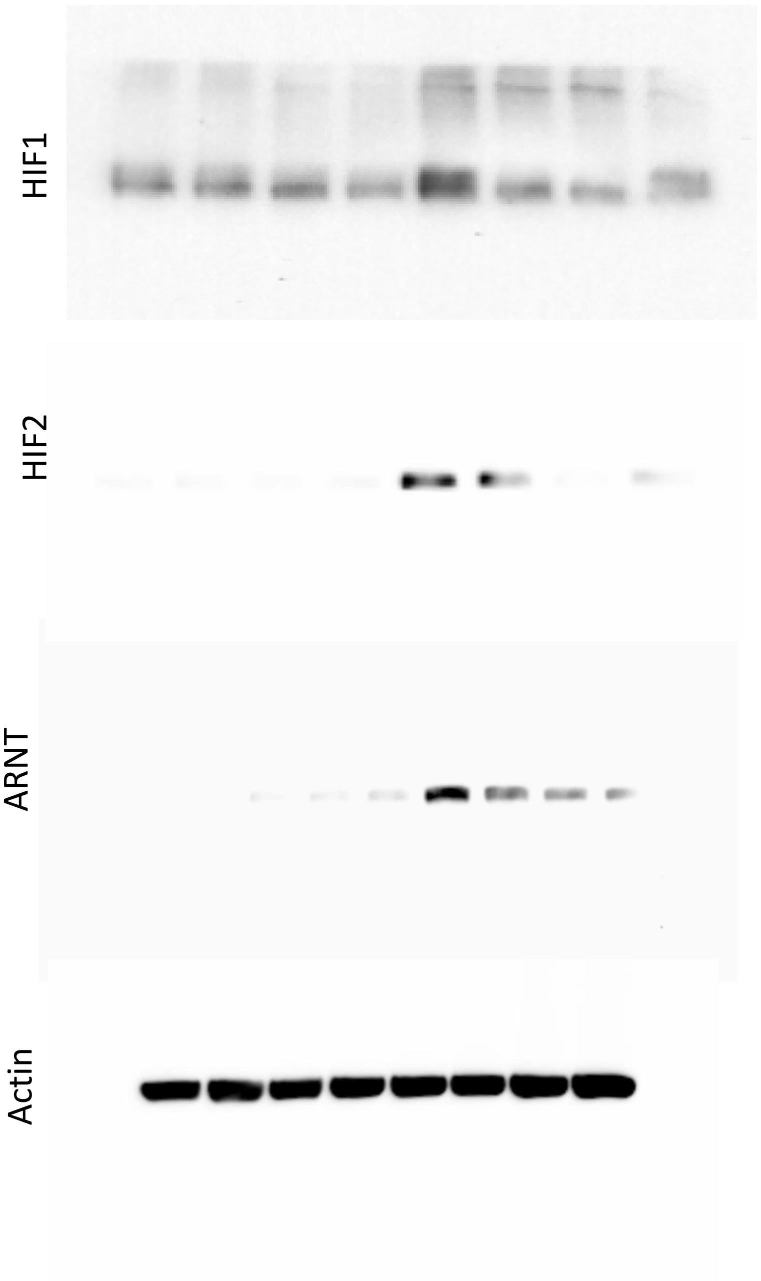


Figure 4B: SUM159 uncropped

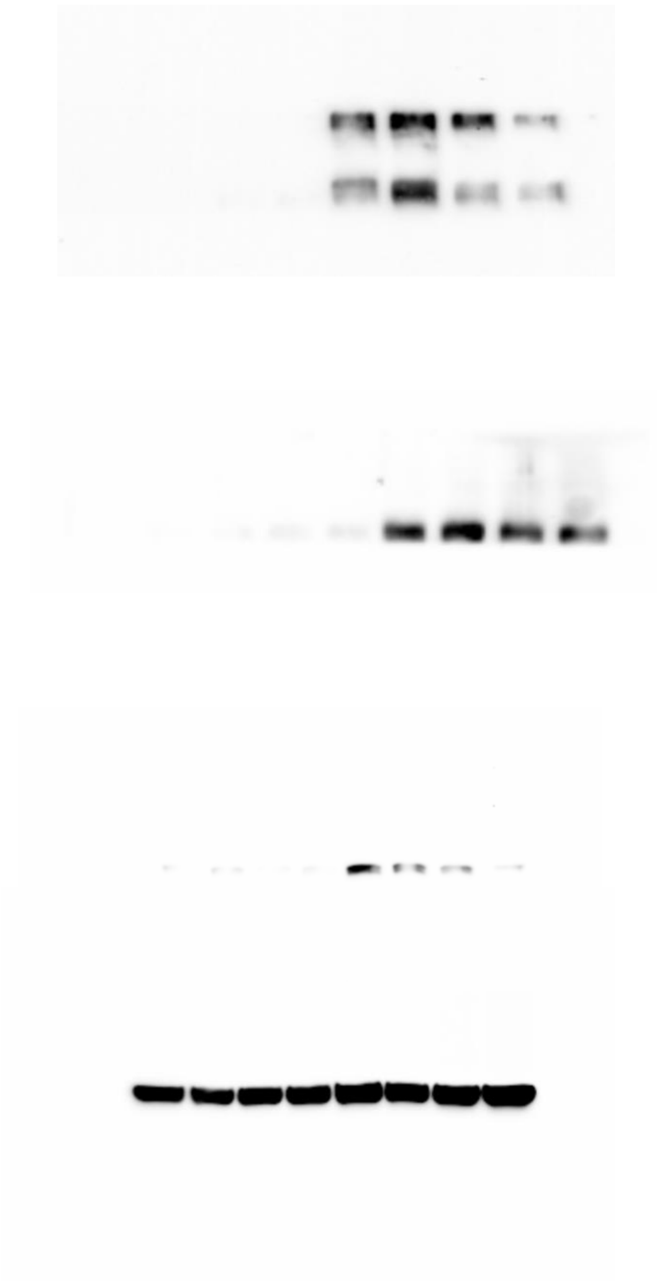

Supplement: Supplementary file 1 [file cancers-15-01330-s001.zip › Supplementary S1 Uncropped immunoblots for Figures 4g,h, 5, and S3c.pdf]
