# Supplementary material for: Mebendazole Treatment Disrupts the Transcriptional Activity of Hypoxia-Inducible Factors 1 and 2 in Breast Cancer Cells
Source: Cancers (Basel). 2023 Feb 20;15(4):1330. doi: 10.3390/cancers15041330 (PMC9954103; doi:10.3390/cancers15041330)
Supplement: Supplementary file 1 [file cancers-15-01330-s001.zip › Supplementary S2 PDX tumors stained for CA9 and quantified using Image J as presentedin Figure 6D.pdf]

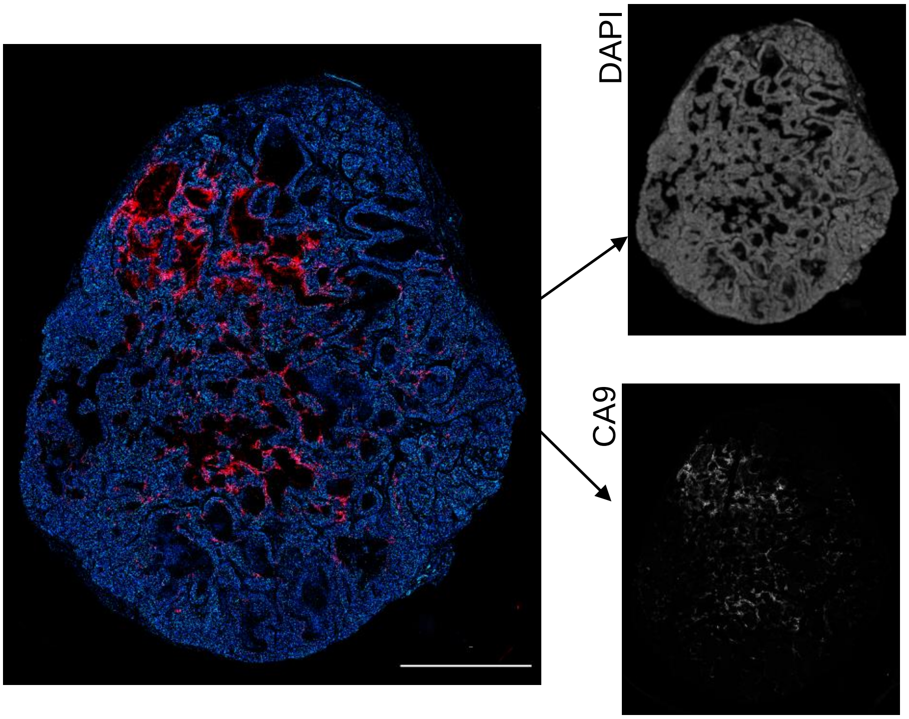

CA9 and DAPI were thresholded manually
to identify % positive areas using IMAGEJ
%CA9 = CA9 Threshold area / DAPI
Threshold area

Mouse ID:

#353 #383 #386 #397 #384

DAPI + CA9

CA9

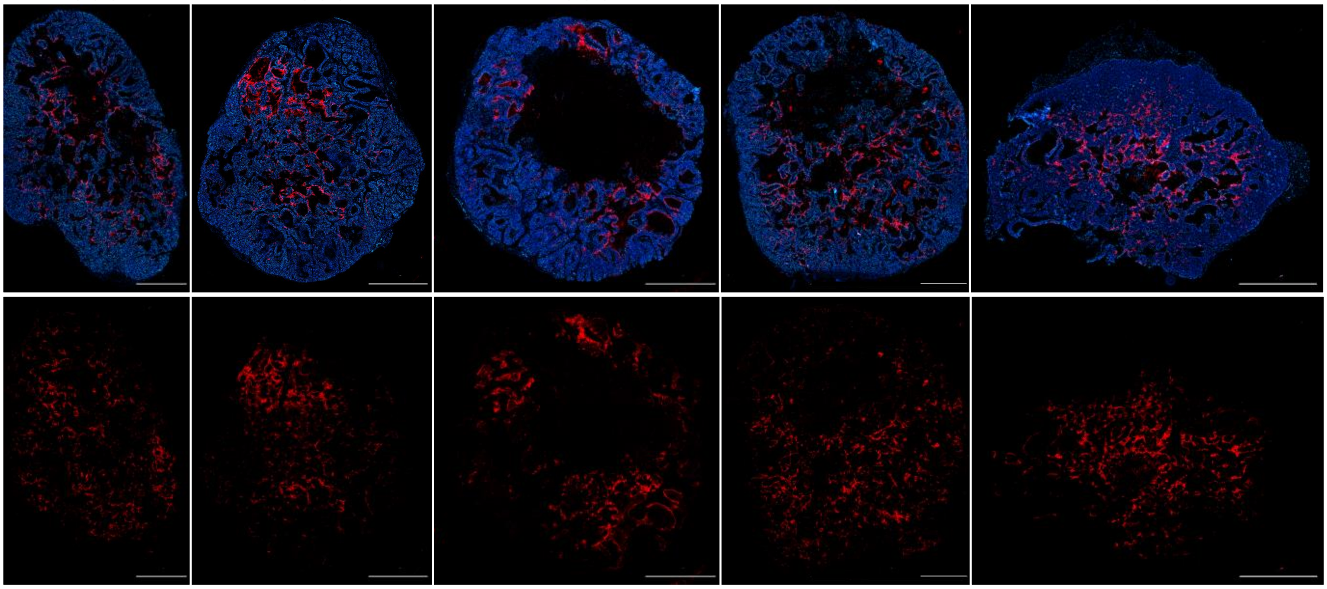

% CA9 Score	#353	#383	#386	#397	#384
	25.64	20.59	16.03	22.42	26.98

#389 #390 #395

DAPI + CA9

CA9

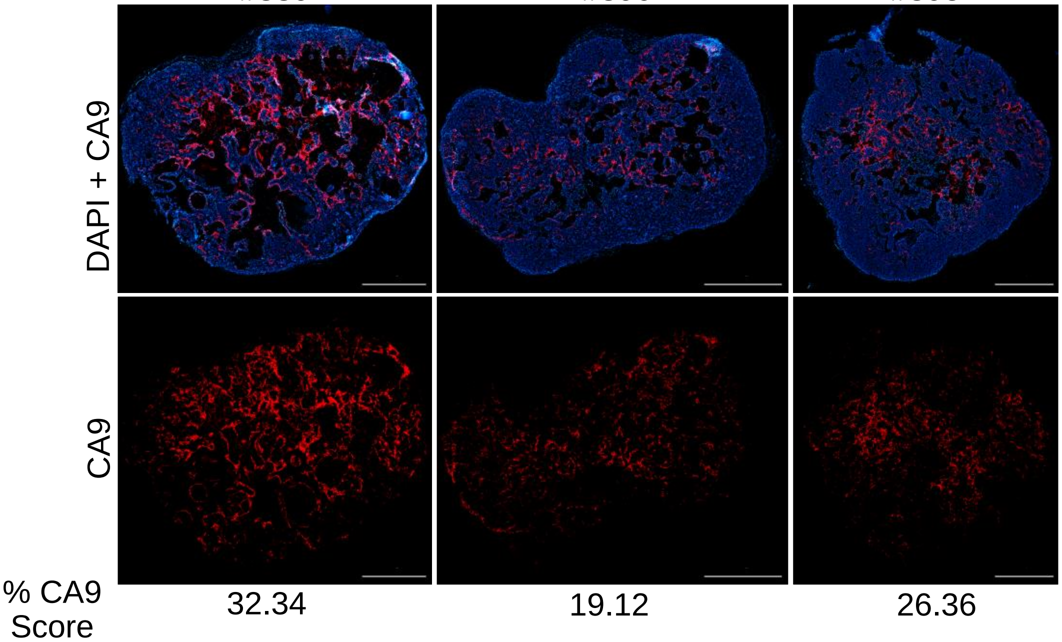

% CA9 Score	#389	#390	#395
	32.34	19.12	26.36

N=8
Vehicle treated mice
Scale bar = 3mm

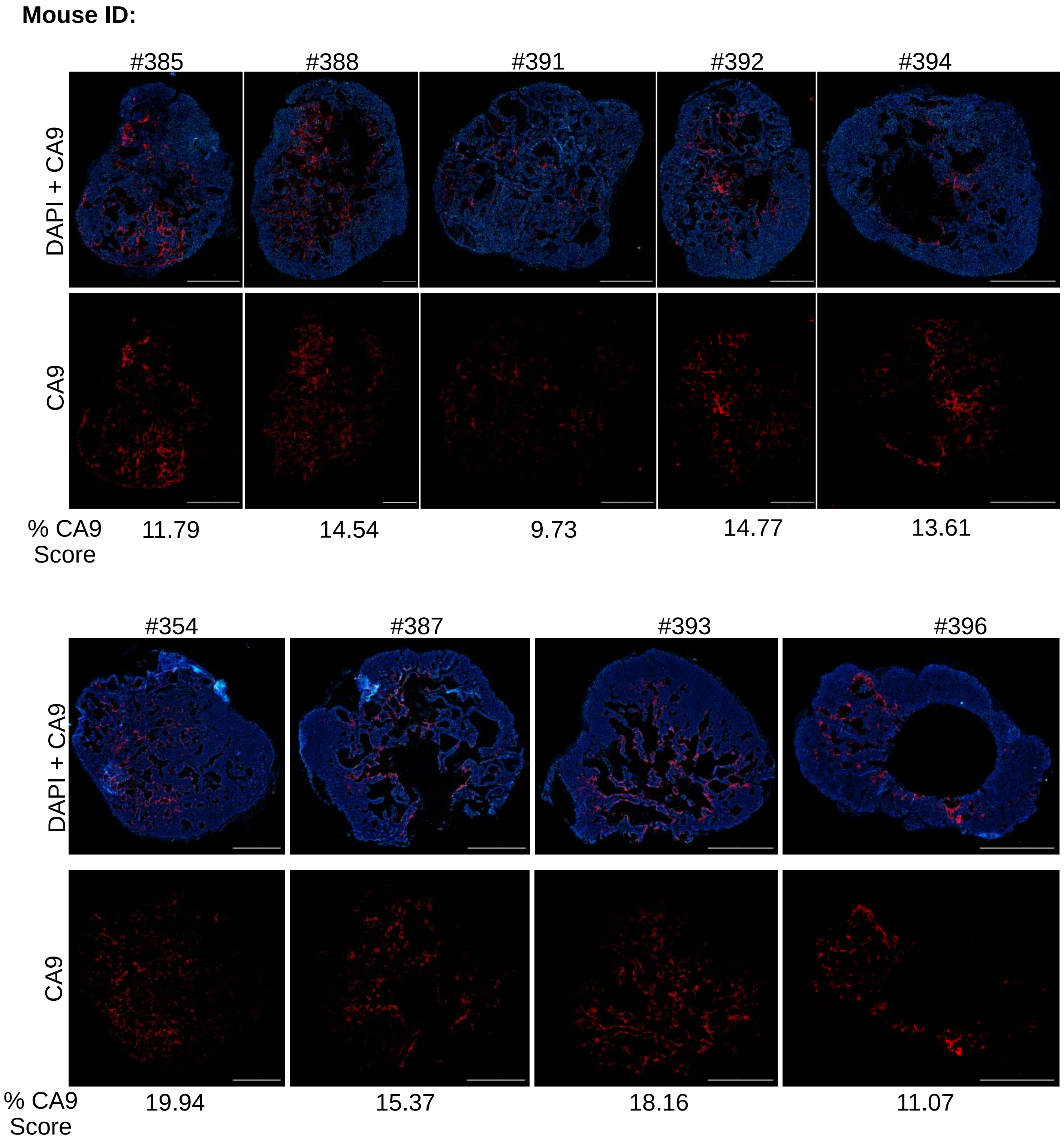

N=9
MBZ treated mice (30mg/kg)
scale bar = 3mm
